# Diversity of trematodes from the amphibian anomaly P hotspot: Role of planorbid snails

**DOI:** 10.1371/journal.pone.0281740

**Published:** 2023-03-29

**Authors:** Anton O. Svinin, Igor V. Chikhlyaev, Ivan W. Bashinskiy, Vitaly V. Osipov, Leonid A. Neymark, Alexander Yu. Ivanov, Tamara G. Stoyko, Polina I. Chernigova, Polina K. Ibrogimova, Spartak N. Litvinchuk, Oleg A. Ermakov

**Affiliations:** 1 Institute of Environmental and Agricultural Biology (X-BIO), Tyumen State University, Tyumen, Russia; 2 Institute of Ecology of Volga River Basin, Samara Federal Research Scientific Center RAS, Togliatti, Russia; 3 Laboratory of Ecology of Aquatic Communities and Invasions, A.N. Severtsov Institute of Ecology and Evolution, Russian Academy of Sciences, Moscow, Russia; 4 Privolzhskaya Lesosteppe State Nature Reserve, Penza, Russia; 5 Department of Zoology and Ecology, Penza State University, Penza, Russia; 6 Institute of Cytology, Russian Academy of Sciences, St. Petersburg, Russia; Universidade Federal de Minas Gerais, BRAZIL

## Abstract

Trematode infection of the second intermediate hosts can lead to changes in their fitness and, as a result, a change in the invasion rate of animal communities. It is especially pronounced during the invasion of parasite species that reduce activity due to the manipulation of hosts through the changes of their morphology and physiology. One of these cases is an anomaly P syndrome hotspot found in some populations of water frogs and toads in Europe caused by the trematode *Strigea robusta* metacercariae. The occurrence of pathogen and their participation in ecosystems are intrigues questions in the anomaly P phenomenon, as well as the role of planorbid snails that serve as the first intermediate hosts for many trematode species. Herein, we focused on trematodes spectra from planorbid snails and amphibians from the anomaly P hosts with the aim to undetected interactions between the pathways of parasites. Emerging cercariae of 6802 planorbid snails of dominant species (*Planorbarius corneus*, *Planorbis planorbis*, and *Anisus* spp.) were detected by both morphological and molecular methods in seven waterbodies in Privolzhskaya Lesostep Nature Reserve (Russia). A total of 95 sequences of 18 species were received, and 48 sequences were unique and did not present in any genetic databases. The 18 species of trematodes from snails and 14 species of trematodes from amphibian hosts (*Pelophylax ridibundus*; Ranidae; Anura) were detected. Three species (*Echinostoma nasincovae*, *Tylodelphys circibuteonis* and *Australapatemon burti*) was new for the trematode fauna of the Middle Volga River region and Russia as a whole. Eleven species of parasitic flatworms have amphibians in their life cycles and nine species used amphibians as metacercariae hosts: *Echinostoma nasincovae*, *E*. *miyagawai*, *Echinoparyphium recurvatum*, *Tylodelphys circibuteonis*, *Neodiplostomum spathula*, *Paralepoderma cloacicola*, *Macrodera longicollis*, *Strigea robusta*, and *Strigea strigis*. The occurrence of trematode species from planorbid mollusks and frogs were compared.

## Introduction

Trematodes (Digenea) as a group of parasitic flatworms play an important role in ecosystems: their larval stages can account for a significant percentage of a total biomass [1], have an impact on the demography of their host populations due to decrease of host survival rates and reproductive success [2], play an important role in the functioning of food webs [3, 4]. Ecology and evolution of host-parasite interactions was an aim in a lot of parasitological research [5, 6]. Special attention has manipulative parasites due to their capability to change host behavior, morphology, and physiology [7, 8]. However, the ecology of manipulative parasites and hosts interactions are on the focus of many parasitologists. Effects of biotic and abiotic factors can change the intensity of invasion and lead to changes in the phenotype or behavior of host [2]. Such changes can be driven by temperature [9], landscapes features [10], presence of certain microbiomes in parasites [11], a synergistic effect of several parasites [12], host and parasite diversity [13], etc.

Diversity of trematodes in various ecosystems in Europe has been aimed by numerous parasitological studies [14–18]. The diversity of trematodes in the ecosystem can be estimated by studying larval stages of trematodes, which parasitize in mollusks as the first intermediate hosts [14, 16–21]. Such studies offer several advantages for describing hidden diversity with non-invasive methods. Due to identification of cercariae by morphology sometimes can be partially limited, combination of morphological diagnostics with molecular analysis can be used for better diagnostics of species [18]. Detection of trematodes from mollusks hosts allow to detect host-parasites interactions, its participation in the functioning of ecosystems, pattern of the spread of trematodoses [18, 20, 21].

**Fig 1:**
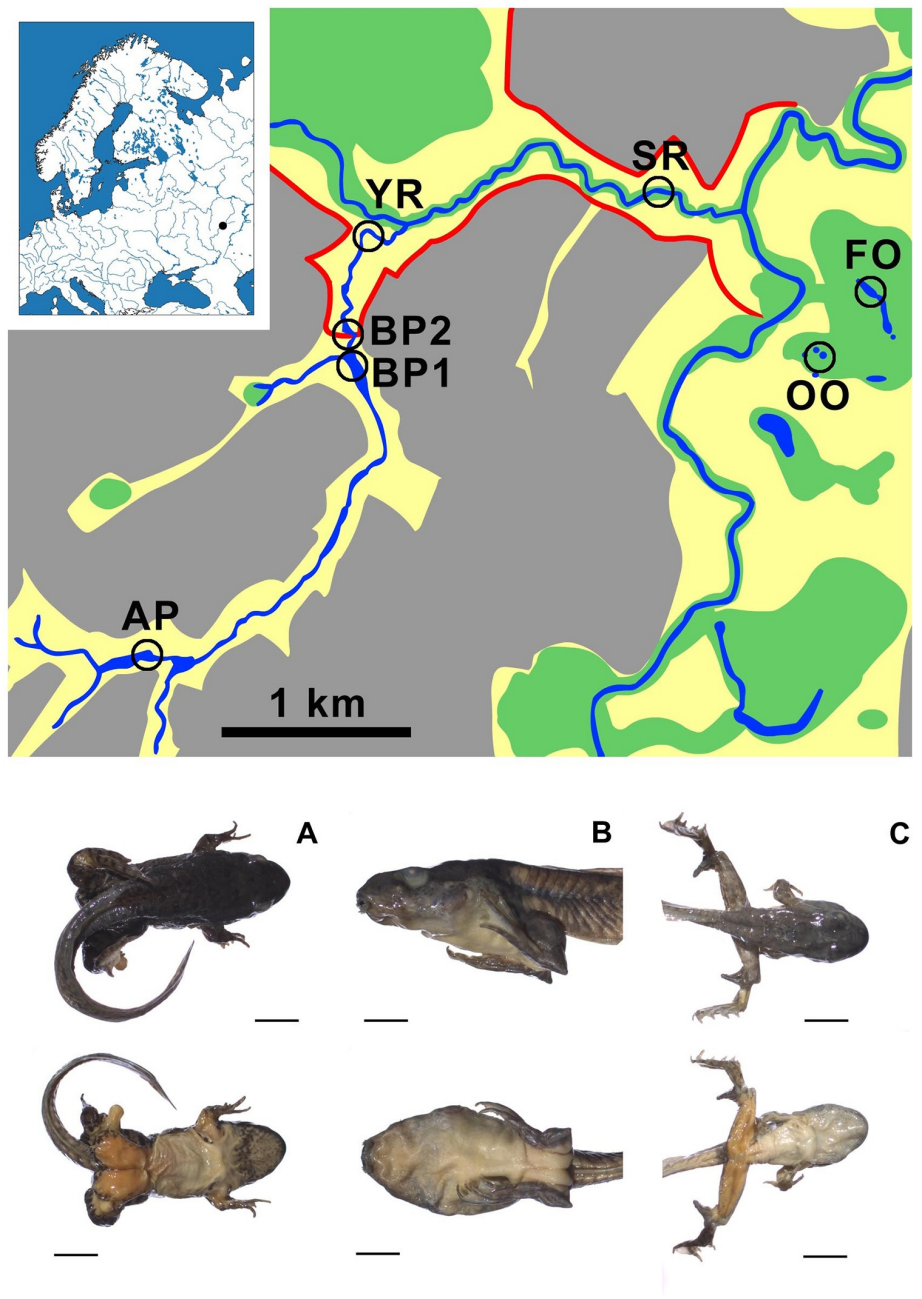
Sampling locations in Ostrovtsovskaya Lesostep’. Small rivers Yuzhnaya (YR) and Selimutka (SR), the floodplain oxbows located in open landscapes (OO) and in the forest (FO), the active beaver pond (BP1) and the drained beaver pond (BP2), and the anthropogenic pond (AP). A, B, C–severe forms of the anomaly P syndrome in *Pelophylax ridibundus* inhabited Ostrovtsovskaya Lesostep’. Scale bar (A–C) is mm.

The global decline of amphibian populations is one of the key problems for biodiversity conservation, and its causes actively discussed by the world scientific community [22–31]. Among the factors that negatively affect amphibian populations and lead to decline are the following: destruction and chemical pollution of habitats, predation by introduced species, exposure to ultraviolet radiation, fungal infections and parasites, etc. [22–24, 30–32]. In Europe, several hotspots are known in which trematodes change the morphology of second intermediate hosts [33]. One of these cases is the anomaly P syndrome hotspot found in some populations of water frogs and toads. The anomaly P includes various deformities of hindlimbs and forelimbs: i.e., flexion of hindlimbs, brachymely, polydactyly, various bone spines, tumor-like formation in inguinal region, and sometimes additional tiny limbs (Fig 1) in tadpoles and metamorphs [33–36]. The anomaly was found to be caused by the trematode *Strigea robusta* (Szidat, 1928) metacercariae [37]. It can lead to a decrease in the survival of amphibian populations, as well as to their extinction [32, 36]. One of the interesting questions left behind the scene is the infection of amphibians from the anomaly P hotspot with other trematode species. Therefore, the aim of present paper is to identify trematodes species composition in snails and frogs of the field sites using morphological examination and molecular analysis. Here, we provide an annotated list of the trematodes in habitats with a high frequency of frogs with the anomaly P syndrome, and discuss their potential effects on amphibian communities and the role of planorbid snails in trematodes pathways.

## Materials and methods

### Sampling of mollusks

We studied waterbodies in the Ostrovtsovskaya Lesostep’ (52.8183° N, 44.4545° E), the part of the nature reserve “Privolzhskaya Lesostep”, Penza region, Russia [38]. For all tasks (abundance of mollusks and screening for cercariae emergence) we investigated seven water objects to reflect all typical aquatic habitats in the forest-steppe landscapes of this part of the nature reserve: small rivers Yuzhnaya (YR) and Selimutka (SR); the floodplain oxbows located in open landscapes (OO) and in forest (FO); the active beaver pond (BP1) and the drained beaver pond (BP2), and the anthropogenic pond (AP) (Fig 1). The description of local biotopes was published previously [39, 40].

### Abundance of freshwater mollusks

#### Sampling by bottom scrapper (method A)

In the summer months of 2016 and 2017, freshwater planorbid snails from two waterbodies (OO and FO) were sampled for the estimation of abundance and taxonomical composition. Mollusks were sampled according to standard methods [40, 41] in littoral zone from the depth of 0.25–0.60 m, manually using a hydrobiological bottom scrapper (width 0.16 m) from the area of 0.48 m^2^. Total amount of samples was 78. The abundance of mollusks was calculated per cubic meter of the bottom and presented in ind./m^3^ (method A, Table 2). The data was partly published in our previous studies on mollusks [39–41]; however, we have completed here our results with sampling by a dip net.

#### Sampling by dip net (method B)

Additionally, in the summer months of 2019 and 2021, in each water body (out of seven), a 1 m^2^ area was demarcated one meter from the shore, and was completely surveyed by sweeping the water column, soil, and vegetation thickets with a dip net (mesh size 3 mm). Mollusks were fixed in 70% ethanol [42] and did not study for trematode invasion. The mollusk screening for cercariae emergence was done in separate samples with other (alive) specimens. The result of the method B was presented in percentage of species out of a total number of planorbid snails in the samples from a waterbody. Total amount of samples was 26.

Finally, a total number of samples made by two methods was 104. Identification of mollusks species was made by TGS in the laboratory of Department of Zoology and Animal Ecology (Penza State University, Penza, Russia) according to determination tables [42, 43].

### Sampling and screening of snails for emerging cercariae

For cercariae detection, alive planorbid snails were caught manually and using hydrobiological nets. The freshwater planorbid snails *Planorbarius corneus* (Linnaeus, 1758), *Planorbis planorbis* (Linnaeus, 1758) and species of *Anisus* Studer, 1820 are prevalent in Ostrovtsovskaya Lesostep’ ([40, 41] and this study) thus they were chosen for screening of cercariae (Table 1). A total of 6802 snails were examined: 4621 *Pl*. *corneus* (S1 Table), 2095 *P*. *planorbis* (S2 Table), and 86 *Anisus* spp. (S3 Table). The snails were transferred into small glass containers (200 ml) filled with 50–100 ml of fresh water. The emergence of cercariae was stimulated by heat and light of an incandescent lamp for 1‒2 h [14, 16]. The initial identification of cercariae was carried out according to morphology [14, 44]. Neutral red stain was used for vital staining. In the field laboratory, we used a Zeiss Primo Star microscope (Carl Zeiss AG, Oberkochen, Germany); in laboratory examinations, cercariae were identified using a microscope Zeiss Axio Imager 2 (Carl Zeiss AG, Oberkochen, Germany).

**Table 1:** Primers used for amplification and sequencing of nuclear and mitochondrial DNA fragments of genes in trematodes examined.

Locus	Primer name	Sequence (5ʹ – 3ʹ)	Reference
ITS2	3S	GGT ACC GGT GGA TCA CGT GGC TAG TG	[51]
	ITS 2.2	CCT GGT TAG TTT CTT TTC CTC CGC	[52]
28S rRNA	dig12	AAG CAT ATC ACT AAG CGG	[53–55]
	1500R	GCT ATC CTG AGG GAA ACT TCG	
COI	JB3	TTT TTT GGG CAT CCT GAG GTT TAT	[51]
	JB4.5	TAA AGA AAG AAC ATA ATG AAA ATG	
	CO1-R trema	CAA CAA ATC ATG ATG CAA AAG G	[56]

### Frog sampling and helminthological survey

A total of 15 adult and subadult marsh frogs (*Pelophylax ridibundus* (Pallas, 1771)) were used in helminthological analysis. Identification of this species from the ponds was made in previous studies by molecular methods (DNA flow cytometry and multiplex PCR) [45]. Seven of them were dissected after fixation, thus we calculated the indices of parasite ability for eight not-fixed animals only. Trematodes were fixed with 70% ethanol and stained with alum carmine and mounted in Canadian balsam. A dimethyl phthalate solution was used to enlighten flatworms. The species of helminths were identified according to field guides [46, 47]. We had taken additional samples of metacercariae from 13 tadpoles and juveniles with polydactyly and severe cases of the anomaly P (tadpoles and juvenile frogs) for subsequent molecular analyses. All procedures with animals were approved by the Mari State University Ethics Committee (Yoshkar‐Ola, Russia). Mollusks after screening returned to natural habitats.

### Molecular identification of trematodes

The DNA was extracted from trematode cercariae and metacercariae by the standard salt-extraction method [48] combined with lysis by proteinase K. Fragments of 28S rRNA, ITS2 (internal transcribed spacer 2) and COI (cytochrome-c-oxidase subunit I) genes were used for identification of trematode species [49, 50] (Table 1). Primers for markers used presents in Table 1 [51–56].

The PCR reaction mixture (25 μL) contained 50–100 ng of DNA, 0.5 μM of each primer, 0.2 mM dNTPs, 1.5 mM MgCl_2_, 2.5 μL 10× PCR buffer (10 mM Tris–HCl, pH 8.3, 50 mM KCl), and 2 units of Taq polymerase (Thermo Scientific). The thermocycling profile of PCR amplification followed those of Tkach et al. [53–55] for 28S rRNA, Bowles et al. and Hugall et al. [51, 52] for ITS2, and Bowles et al. and Miura et al. [51, 56] for COI. The PCR fragments were prepared for sequencing by elution with a high-salt solution from a 6% polyacrylamide gel. Sequencing was performed on an ABI 3500 automatic sequencer (Applied Biosystems) using the BigDye^®^Terminator 3.1 (Applied Biosystems) kit and the same primers that were used for amplification.

The sequences were aligned and edited manually in the Chromas v. 2.5.1 (Technelysium Ltd., Australia). Screening of the primary sequences most similar to those of our sequences was performed with the BLAST algorithm [57]. The GenBank NCBI accession numbers of our sequences are present in S4 Table. Maximum Likelihood (ML) used to estimate phylogenetic tree. Best-fit models of evolution determined in IQ-TREE [58] using the Bayesian information criterion (BIC) implemented in ModelFinder [59]. K3Pu+F+I+G4 was the best-fit model of evolution for COI gene fragment, and TVM+F+I+G4 was the best-fit model of evolution for 28S rRNA and ITS2. The ML analyses were performed using the IQ-TREE webserver [60] with 1,000 UFBoot iterations [61, 62]. Data were visualized and edited with FigTree v.1.4.3.

## Results

### Prevalence of planorbid snails and infection rates

In waterbodies examined, seven species of planorbid snails were found: *Bathyomphalus contortus* (Linnaeus, 1758), *Bathyomphalus crassus* da Costa, 1778, *Pl*. *corneus*, *P*. *planorbis*, *Anisus spirorbis* (Linnaeus, 1758), *Anisus vortex* (Linnaeus, 1758), and *Anisus vorticulus* (Troschel, 1834) (Table 2). The dominant species in the lentic waterbodies were *Pl*. *corneus* (11.7–92.3%), *P*. *planorbis* (5.8–82.3%), *Anisus vorticulus* (50.0%), *Bathyomphalus crassus* (23.6%) and *Anisus vortex* (16.3%) (Table 2). In the lotic waterbodies, the dominant species of planorbid snails was *Bathyomphalus contortus* (100%). Because reproduction and development of amphibians occur in stagnant water, infection of snails has been studied only from lentic water bodies.

**Table 2:** Prevalence of planorbid snails in waterbodies examined.

Waterbodies	% Planorbidae^a^	Species	Method A^b^, ind./m^3^	Method B^c^, %	% inv.^d^
Selimutka River	12.5%	*Bathyomphalus contortus*	–	100	–
Yuzhnaya River	8.0%	*Bathyomphalus contortus*	–	100	–
Open Oxbow	44.6%	*Planorbarius corneus*	1.1–1.7	15.7	11.2
		*Planorbis planorbis*	3.6–10.9	82.3	12.7
		*Anisus spirorbis*	0.0–0.4	2.0	3.3
Forest Oxbow	54.3%	*Planorbarius corneus*	0.4–0.5	11.7	45.6
		*Planorbis planorbis*	1.7–2.2	48.3	13.0
		*Anisus vortex*	0.0–1.45	16.3	3.8
		*Bathyomphalus crassus*	0.6–1.2	23.6	–
Beaver Pond 1	19.9%	*Planorbarius corneus*	–	92.3	28.3
		*Planorbis planorbis*	–	5.8	18.8
		*Anisus vorticulus*	–	1.9	0.0
Beaver Pond 2	23.5%	*Planorbarius corneus*	–	91.7	61.0
		*Planorbis planorbis*	–	0.0	6.7
		*Bathyomphalus contortus*	–	8.3	–
Anthropogenic Pond	75.0%	*Planorbarius corneus*	–	50.0	29.8
		*Anisus vorticulus*	–	50.0	–

^a^ percentage of planorbid snails out of a total number of mollusks;

^b^ abundance of mollusks detected by method A;

^c^ percentage of species out of a total number of planorbid snails in a waterbody detected by method B (see Materials and methods);

^d^ percentage of infected mollusks.

The infection rates of mollusks varied in various waterbodies. A total number of infected *P*. *planorbis* was 6.7–18.8%, while among *Anisus* spp. were infected 0–3.8% of snails; *Pl*. *corneus* was the most infected species and 11.2–61.0% of mollusks had emerging cercariae (Table 2). Among waterbodies, the most infected snails were found in Beaver ponds (*P*. *planorbis*, *Pl*. *corneus*); while in Open Oxbow intensity of trematode invasion was the lowest (11.2% in *Pl*. *corneus*).

### Diversity of cercariae emerged from planorbid snails

**Fig 2:**
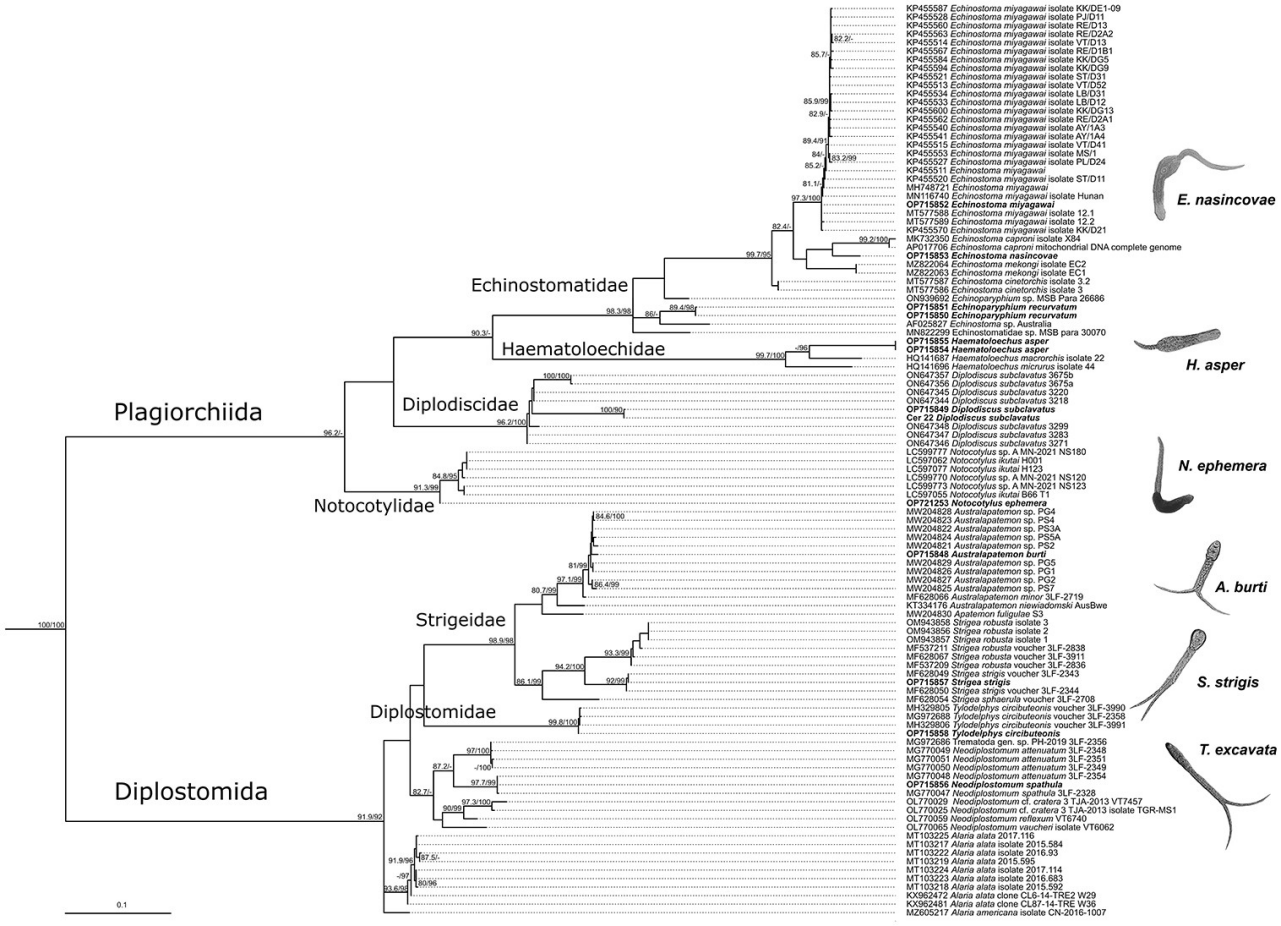
Phylogenetic relationship of trematodes based on COI sequences. Maximum-likelihood phylogenetic tree of trematode species inferred using IQ-TREE with 1,000 SH-like approximate likelihood ratio test (SH-aLRT) and ultra-fast bootstrap (UFboot) replicates each. AM913860, AM913862 and AM913865 *Polystoma* are used as an outgroup. iNumbers at nodes indicate SH-aLRT support (≥80%)/UFboot support (≥95%); values less shown with “-”.

**Fig 3:**
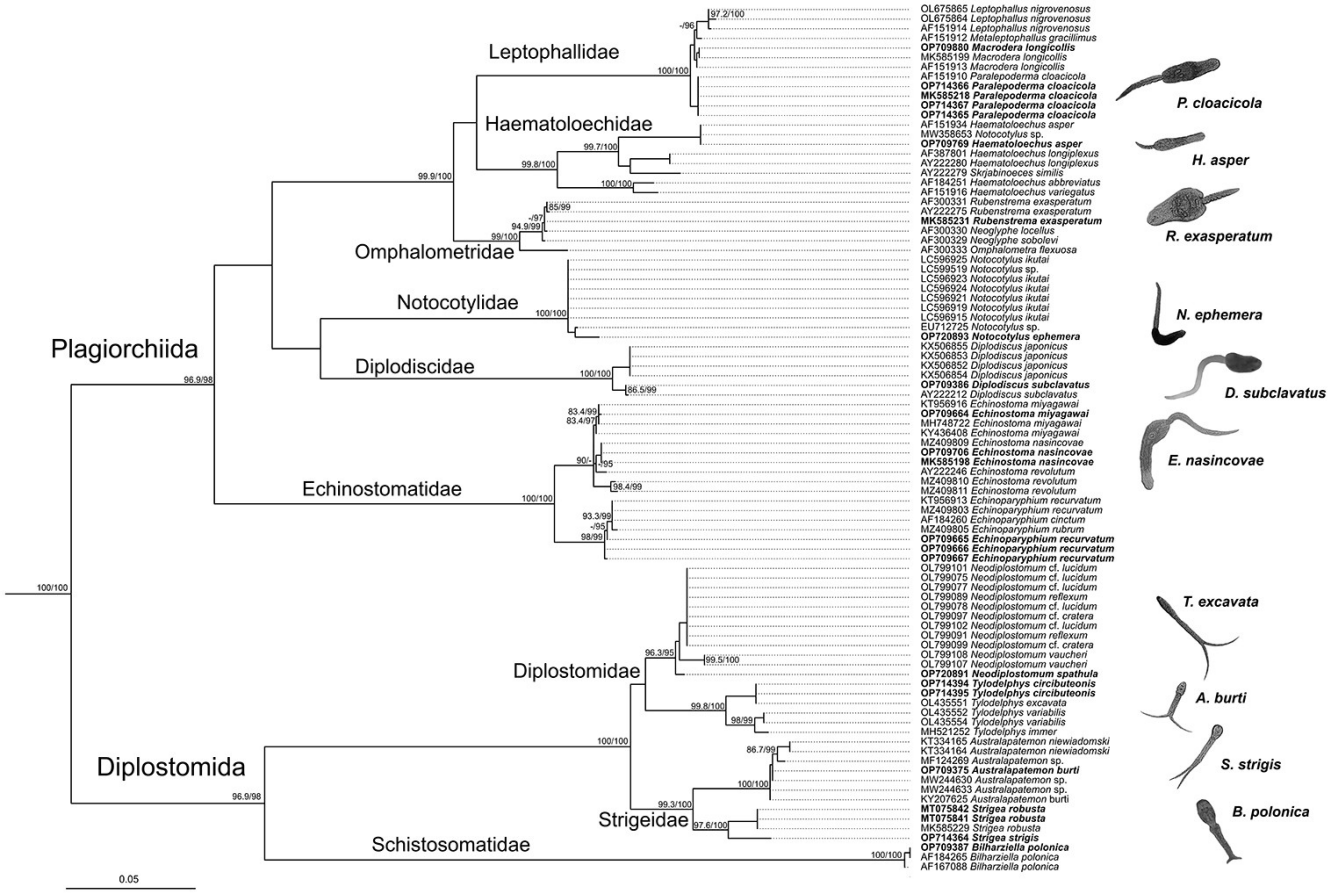
Phylogenetic relationship of trematodes based on 28S rRNA sequences. Maximum-likelihood phylogenetic tree of trematode species inferred using IQ-TREE with 1,000 SH-like approximate likelihood ratio test (SH-aLRT) and ultra-fast bootstrap (UFboot) replicates each. AY222162, MK387333 *Aspidogaster* and AY222163 *Multicalyx* are used as an outgroup. Numbers at nodes indicate SH-aLRT support (≥80%)/UFboot support (≥95%); values less shown with “-”.

A total of 18 species of trematodes, including 11 species for *P*. *planorbis*, 8 species for *Pl*. *corneus*, and two species for *Anisus vortex* were found (Tables 3 and 4; S3 Table). The trematode species belonged to ten families from two suborders, Diplostomida (3 families) and Plagiorchiida (7 families): Diplostomidae Poirier, 1866; Strigeidae Railliet, 1919; Schistosomatidae Stiles et Hassall, 1898; Paramphistomidae Fischoeder, 1901; Echinostomatidae Looss, 1902; Diplodiscidae Cohn, 1904; Notocotylidae Lühe, 1909; Haematoloechidae Freitas et Lent, 1939; Omphalometridae Odening, 1960 and Leptophallidae Dayal, 1938. Phylogenetic analysis based on 28S rRNA fragment and mitochondrial COI (Figs 2 and 3) supported traditional division into families and suborders.

**Table 3:** Occurrence of trematode species in *Planorbarius corneus* (*n* = 4621).

Species of trematodes	Open	Beaver	Beaver	Forest	Anthropogenic	Total
	Oxbow	Pond 1	Pond 2	Oxbow	Pond	
	(*n* = 1206)	(*n* = 690)	(*n* = 2430)	(*n* = 114)	(*n* = 181)	(*n* = 4621)
*Australapatemon burti*	-	-	0.04	-	-	0.01
*Bilharziella polonica*	0.4	0.1	0.3	4.4	0.6	1.2
*Echinostoma nasincovae*	0.4	-	0.1	-	-	0.1
*Haematoloechus asper*	2.3^a^	1.0	0.1	0.9	2.2	1.3
*Notocotylus ephemera*	0.5	5.5	1.8	0.9	6.1	3.0
*Rubenstrema exasperatum*	7.2	20.6	58.2	37.7	19.9	28.7
*Strigea robusta*	0.2	0.4	0.2	-	1.1	**0.4**
*Tylodelphys circibuteonis*	0.2	0.6	0.2	1.8	-	0.5

^a^ % of a total number of *Planorbarius corneus* mollusks.

**Table 4:** Occurrence of trematode species in *Planorbis planorbis* (*n* = 2095).

Species of trematodes	Open	Beaver	Beaver	Forest	Total
	Oxbow	Pond 1	Pond 2	Oxbow	
	(*n* = 978)	(*n* = 64)	(*n* = 570)	(*n* = 483)	(*n* = 2095)
*Australapatemon burti*	2.1	3.1	0.9	1.0	1.8
*Diplodiscus subclavatus*	0.9^a^	-	-	-	0.2
*Echinoparyphium recurvatum*	0.9	-	0.5	0.4	0.5
*Echinostoma revolutum*	0.6	3.1	0.5	0.8	1.3
*Haematoloechus asper*	2.4	7.8	2.6	2.1	3.7
*Macrodera longicollis*	-	-	0.2	-	0.04
*Neodiplostomum spathula*	0.1	-	-	0.4	0.1
*Paralepoderma cloacicola*	3.4	1.6	0.5	0.6	1.5
*Stichorchis subtriquetrus*	0.4	1.6	1.2	0.2	0.9
*Strigea robusta*	-	1.6	-	-	**0.4**
*Strigea strigis*	-	-	0.2	-	0.04

^a^ % of a total number of *Planorbis planorbis* mollusks.

Trematode sequences obtained show high similarity with the available sequences in the GenBank NCBI (Figs 2 and 3; S1 Fig, S4 Table). We received 95 sequences of 18 species, and 48 sequences of them were unique. For each species, we have received unique sequences for at least one locus of three (except *Notocotylus ephemera*).

Family Diplostomidae was presented in our studies by two species: *Tylodelphys circibuteonis* Odening, 1962 and *Neodiplostomum spathula* (Creplin, 1829). Morphological examination of diplostomid cercariae from *Pl*. *corneus* allowed us to identify cercariae as *Tylodelphys excavata* (Rudolphi, 1803). However, Heneberg and Sitko [63] identified a previously unknown species complex in *T*. *excavata* sensu lato and redescribed cryptic species *T*. *circibuteonis* Odening, 1962 from the white stork, *Ciconia ciconia* (Linnaeus, 1758). According to COI sequences, our *T*. *excavata*-like cercariae have similar sequences to *T*. *circibuteonis* (MH329806, MH329805, and MG972688). According to our ITS2 sequence (OP704224), it is form a clade with *T*. *circibuteonis* (MG972696), however which is poorly supported. Heneberg and Sitko [63] had shown the similarity of ITS2 sequences of *T*. *excavata* (KC685364) from *Pl*. *corneus* with their *T*. *circibuteonis* (MG972696). Achatz et al. [64] represents 28S rRNA sequences of *T*. *excavata* from *Pelophylax ridibundus* that are similar to our sequences (OP714394, OP714395) and is likely to be *T*. *circibuteonis*. If our species is *T*. *circibuteonis*, then we present new information about the life cycle of this species of trematodes (that used the marsh frogs as a second intermediate host), and also describe it for the first time for the fauna of the Volga River region and Russia, in general.

Diplostomid cercariae from *P*. *planorbis* were identified by morphological features as *Neodiplostomum spathula* (Creplin, 1829) La Rue, 1926. According to our sequences of ITS2 and COI, it was identical to sequences of *N*. *spathula* (MG770065) from *Clanga pomarina* in the Czech Republic [65]. We have deposited in GenBank new sequence of 28S rRNA gene fragment for this species.

We identified strigeid cercariae according to morphology as representatives of two genera: *Strigea* Abildgaard, 1790 and *Australapatemon* Sudarikov, 1959. *Australapatemon burti* is extremely similar to *Australapatemon minor* (Yamaguti, 1933) according to sequences of COI, ITS2, and 28S rRNA. The taxonomic status of these species has been discussed [66] and, probably, *A*. *burti* and *A*. *minor* represent the same species with a high intraspecific genetic variability. According to the sequence ITS2, our specimen of *Australapatemon* (OP693490) is identical to *A*. *burti* (JX977787, KU950451). Thus, we follow D. I. Hernández-Mena et al. [67] and O.V. Aksenova et al. [68] and assign our *Australapatemon* to *A*. *burti*.

Species of the genus *Strigea* display a limited number of morphologic differences on larval stages. They have similar flame-cell formula 2[(1 + 1) + (1 + 1 + [1])] = 10, however *S*. *robusta* has spinose entire body, a row of large straight spines on a ventral sucker and caeca terminate at level of posterior margin of ventral sucker [14]. Our molecular analysis revealed two species of *Strigea*: *S*. *robusta* and *S*. *strigis*. The last species observed only once (in the BP2) and can be considered as a rare species in the landscapes examined. *Strigea robusta* had similar sequences of COI and ITS2 with the sequences from GenBank (MF537205, MF537209, and MF537211); however, sequences of 28S rRNA are unique for our recent and previous studies. The teratogenic effect of *S*. *robusta* cercariae from *Pl*. *corneus* inhabited Ostrovtsovskaya Lesostep’ has been repeatedly shown in experiments with laboratory-reared tadpoles of water frogs and toads [37, 69, 70].

The family Schistosomatidae was represented by only one species of trematode in planorbid snails: *Bilharziella polonica* (Kowalewsky, 1895) Looss, 1899. We did not get COI sequence for this species; however, 28S rRNA (OP709387) and ITS2 (OP693618) sequences were similar with known for this species.

The family Leptophallidae includes species parasitized in the grass snake (*Natrix natrix* (Linnaeus, 1758)), as the definitive host, and used anuran amphibians as the second intermediate hosts. Two species of leptophallids (*Paralepoderma cloacicola* and *Macrodera longicollis*) found in *P*. *planorbis* have a limited number of morphological differences on the cercariae stage. Our sequences of COI and ITS2 for *P*. *cloacicola* and ITS2 for *M*. *longicollis* were unique, and sequences of 28S rRNA were identical with those from the paper of Tkach et al. [53]. We were unable to get COI amplification and sequencing for *Macrodera longicollis* using JB3 and JB4.5 primers.

In the family Haematoloechidae, only one species was found on the cercariae stage—*Haematoloechus asper*. Nevertheless, in the helminthological survey (see below) two more species from this family were registered which also choose planorbid snails as the first intermediate hosts. Probably, these species were hidden among the presented *H*. *asper* cercariae and have not been detected using morphological and molecular studies of larvae. The sequence MW358653 from GenBank of 28S rRNA was similar with our *H*. *asper*, but named mistakenly as *Notocotylus* sp.

Identification of species from the family Omphalometridae had some difficulties. Our sequences of 28S rRNA (MK585231) for species of the *Rubenstrema exasperatum* / *Neoglyphe locellus* complex have two variable nucleotide substitutions: one (A675G) distinguishes from *Rubenstrema exasperatum* (Rudolphi, 1819) (GenBank NCBI No. AF300331 and AY222275) and second (A1092G) from *Neoglyphe locellus* (Kossack, 1910) (GenBank No. AF300329 and AF300330). Sequences of *R*. *exasperatum* and *N*. *locellus* from GenBank NCBI differ only in three positions (two of them described above) [54]. Thus, we have no possibility to identified the species, but morphologically it is closer to *R*. *exasperatum* due to their large size of stylet (35 μm long) [14].

We had no found sequences of *Notocotylus ephemera* (Nitzsch, 1807) Harwood, 1939 in the GenBank NCBI, however, according to the morphology of larvae and the choice of host [14], we can safely attribute it to the species. Thus, we have replenished the genetic database for all three markers of the species.

*Diplodiscus subclavatus* (Pallas, 1760) from waterbodies studied by us had a unique COI sequence (OP715849) compared to the species genetic lineages from more western parts of Europe (ON647344—ON647348; ON647356; ON647357) [71]. Probably, under the name *Diplodiscus subclavatus* sensu lato two species are hidden, one of which parasitizes European amphibians, and another one found in amphibian populations from the Volga River basin. According to 28S rRNA data, our sequence is very close (99.9%, 1 substitution out of 1277 b.p.) to that of *D*. *subclavatus* (AY222212) presented by P.D. Olson et al. [72]. Our sequences of ITS2 are identical (100%) to *D*. *mehrai* Pande, 1937 (MW000971 and MW000970) from Danish freshwater systems [18]. However, authors classified their *Diplodiscus* as *D*. *mehrai* by mistake (according to BLAST similarity) and it should be identified as *D*. *subclavatus*. It should be noted that two closely related species can be combined under this name. *Diplodiscus subclavatus*, which was described by P. S. Pallas (1760) from Western Europe, can probably be attributed to *D*. *subclavatus*; while our species (it is possible that the species occur sympatrically) is a new undescribed taxon. Further taxonomic research may help clarify this issue.

According to COI sequence (OQ352877; Cer25 voucher) identity to *Stichorchis subtriquetrus* haplotype Hap7 (OL451236), we determine the paramphistomid cercariae as the trematode species *Stichorchis subtriquetrus*.

The family Echinostomatidae consists of three species: *Echinoparyphium recurvatum* (Linstow, 1873), *Echinostoma revolutum* (Fröhlich, 1802) Looss, 1899, and *Echinostoma nasincovae* Faltýnková, Georgieva, Soldánová et Kostadinova, 2015. The last species was described from *Pl*. *corneus* inhabited waterbodies of Czech Republic based on morphological and molecular analyses [73–75]. Our sequences of *Echinostoma* from *Pl*. *corneus* were identical to the *E*. *nasincovae* sequence (MZ409809 for 28S rRNA).

### Infection prevalence in mollusks

*Planorbarius corneus* was infected in 41.5%, while *P*. *planorbis* was infected in 11.1% (S1 and S2 Tables). The most prevalent species was the trematode *Rubenstrema exasperatum* (28.7%; see Table 2) detected from *Pl*. *corneus*. In *P*. *planorbis* the most dominant species of trematodes was *Haematoloechus asper* (3.7%).

Two species of trematodes (*Haematoloechus asper* Looss, 1899 and *Strigea robusta*) were found in both species of mollusks, while other species were specific to one host. A specimen of mollusk *Pl*. *corneus* was occasionally infected (one snail out of 4621, i.e., 0.02%) by *Australapatemon burti* (Miller, 1923).

Seven cases of double invasion were registered (0.11% in *Pl*. *corneus*, 0.09% in *P*. *planorbis*). In the mollusk *Pl*. *corneus*, the trematode *Rubenstrema exasperatum* was found together with *Tylodelphys circibuteonis*, *Notocotylus ephemera* (two cases) or *Bilharziella polonica* (Kowalewski, 1895), and the trematode *Strigea robusta* together with *Haematoloechus asper* (one case). In the snail *P*. *planorbis*, *Diplodiscus subclavatus* coexisted with *Paralepoderma cloacicola* (Lühe, 1909), and the trematode *Australapatemon burti* coexisted together with *Haematoloechus asper*.

Trematode species parasitizing in amphibians have been found in two planorbid mollusks: four species out of eight (50%) in *Pl*. *corneus* and nine species out of 11 (82%) in *P*. *planorbis*. *Strigea robusta* and *H*. *asper* can infect both planorbid mollusks. The prevalence of this species of trematodes in snails was as following: *Diplodiscus subclavatus* occurred in 0.2% of all snails, *Echinoparyphium recurvatum* (Linstow, 1873) in 0.5%, *Echinostoma revolutum* (Fröhlich, 1802) Looss, 1899 in 1.3%, *Echinostoma nasincovae* Faltýnková, Georgieva, Soldánová et Kostadinova, 2015 in 0.1%, *Haematoloechus asper* occurred in 1.3% and 3.7% snails (*Pl*. *corneus* and *P*. *planorbis*, respectively), *Macrodera longicollis* (Abildgaard, 1788) in 0.04%, *Neodiplostomum spathula* (Creplin, 1829) La Rue, 1926 in 0.1%, *Paralepoderma cloacicola* in 1.5%, *Strigea strigis* (Schrank, 1788) Abildgaard, 1790 in 0.04% (the most rare trematode), and *Tylodelphys circibuteonis* in 0.5% of a total number of snails.

The highest Simpson index value was observed in trematode fauna in *Pl*. *corneus* from Beaver Pond 2 (Table 5). The high index value is due to the predominance of *Rubenstrema*, which infects about 60% of snails. A decrease in infestation by this species and an equalization of the abundance of others leads to low Shannon index value in Open Oxbow (Table 5).

**Table 5:** Diversity indices for species of trematodes from Ostrovtsovskaya Lesostep’ waterbodies.

Localities	Simpson index		Shannon index	
	*Planorbarius corneus*	*Planorbis planorbis*	*Planorbarius corneus*	*Planorbis planorbis*
Open Oxbow	0.459	0.221	1.117	1.691
Forest Oxbow	0.689	0.223	0.659	1.662
Beaver Pond 1	0.568	0.182	0.840	1.583
Beaver Pond 2	0.910	0.216	0.246	1.659
Artificial Pond	0.484	-	0.983	-

### Occurrence of *Strigea robusta* infection

The trematode *Strigea robusta* was found in four waterbodies out of five examined: we did not find it in Forest Oxbow only. A total prevalence of *Strigea robusta* in mollusks was the same in *P*. *planorbis* and *Pl*. *corneus* (0.4%). The presence of this parasite in mollusks varies among ponds: 0.2% of *Pl*. *corneus* from Beaver Pond 1 and Open Oxbow, while in Anthropogenic Pond we found 1.6% infected *Pl*. *corneus*.

### Diversity of metacercariae and adult flatworms in frogs

According to our molecular analysis, the trematode fauna of water frog tadpoles in the vicinity of Ostrovtsovskaya Lesostep’ consisted of 14 species of trematodes (Table 6). *Brandesia turgida* (Brandes, 1888), *Paralepoderma cloacicola*, *Macrodera longicollis*, *Opisthioglyphe ranae* (Frӧlich, 1791) were detected with the use of molecular methods (using 28S rRNA and ITS2 markers), other species were identified morphologically. *Opisthioglyphe ranae* was observed in 11 tadpoles, *Paralepoderma cloacicola* in three, and *Macrodera longicollis* in a single (S4 Table).

**Table 6:** Trematodes (adult worms and metacercariae) from the individuals (*n* = 15) of the marsh frog (*Pelophylax ridibundus*) from waterbodies examined.

Species of trematodes	Stage^a^	Localization	Percentage of individuals	Min—max	Abundance Index
*Gorgodera asiatica*	ad.	Urinary bladder	6.67	1	0.07
*Haematoloechus variegatus*	ad.	Lungs	13.33	1–3	0.27
*Skrjabinoeces similis*	ad.	Lungs	6.67	2	0.13
*Brandesia turgida*	ad.	Small intestine	6.67	5	0.33
*Opisthioglyphe ranae*	ad., mtc.	Small intestine	66.67	1–111	16.87
*Pleurogenes claviger*	ad.	Small intestine	13.33	9–58	4.47
*Pleurogenoides medians*	ad.	Small intestine	33.33	1–42	6.00
*Prosotocus confusus*	ad.	Small intestine	53.33	1–72	10.53
*Diplodiscus subclavatus*	ad.	Large intestine	53.33	1–30	5.27
*Paralepoderma cloacicola* + *Macrodera longicollis* ^b^	mtc.	Muscles of the tongue, urinary bladder wall	75.00	4–90	18.63
*Strigea strigis* ^b^	mtc.	Femur muscles	12.50	1	0.13
*Pharyngostomum cordatum* ^b^	mtc.	Femur muscles	12.50	4	0.50
*Tylodelphys excavata* sensu lato ^b^	mtc.	Spinal canal	62.50	2–255	53.63

^a^ ad. = adult worms; mtc. = metacercariae;

^b^ calculation was carried out except fixed amphibians (*n* = 8).

The most prevalent species in post-metamorphic frogs were *Tylodelphys excavata complex* (53.6%), *Paralepoderma cloacicola* (18.6), *Opisthioglyphe ranae* (16.9), *Prosotocus confusus* Looss, 1899 (10.5), and *Pleurogenes claviger* (Rudolphi, 1819) (4.5). We found no statistically significant correlation between abundance of trematodes in snails and frogs (r = 0.50; *p* = 0.391) (Table 7).

**Table 7:** Occurrence (%) of trematodes in the first and second intermediate hosts.

Species of trematodes	First intermediate host	% in planorbid snails (cercariae)	% in frogs (metacercariae and adult worms)
*Gorgodera asiatica*	Bivalvia	-	0.07
*Haematoloechus asper*	*Planorbis planorbis*	1.3	-
	*Planorbarius corneus*	3.7	-
*Haematoloechus variegatus*	*Planorbis planorbis*	-	0.27
*Skrjabinoeces similis*	*Planorbis planorbis*	-	0.13
*Brandesia turgida*	Unknown	-	0.33
*Opisthioglyphe ranae*	*Lymnaea stagnalis*	-	16.87
*Pleurogenes claviger*	*Bythinia tentaculata*	-	4.47
*Pleurogenoides medians*	*Bythinia tentaculata*	-	6.00
*Prosotocus confusus*	*Bythinia tentaculata*	-	10.53
*Diplodiscus subclavatus*	*Planorbis*, *Anisus*	0.2	5.27
*Paralepoderma cloacicola*	*Planorbis planorbis*	1.5	18.63
*Macrodera longicollis*	*Planorbis planorbis*	0.04	-
*Strigea strigis*	*Planorbis planorbis*	0.04	0.13
*Pharyngostomum cordatum*	*Planorbis planorbis*	-	0.50
*Tylodelphys circibuteonis*	*Planorbarius corneus*	0.5	53.63

### Abnormality rates in frogs

Long-term study of the anomaly P hotspot in the population of the marsh frog, *Pelophylax ridibundus* (Pallas, 1771), was conducted during the period of seven years (2016–2022). The abnormality rates in populations were 14.7–36.8% (on average 17.8) for polydactylous specimens and 3.8–10.7% (6.7) for heavy cases of the syndrome (Table 8). Abnormal individuals from Anthropogenic Pond and Beaver Pond 2 were not found, however *Strigea robusta* cercariae were emerged from local snails. The cercariae of this species were not detected from the Forest Oxbow’s mollusks, but the presence of this trematode is not in doubt and its occurrence in mollusks can be less than 0.2%.

**Table 8:** Occurrence (%) of *Strigea robusta* and abnormal individuals of the marsh frog, *Pelophylax ridibundus*, in waterbodies examined.

Waterbodies	Host species	*n*	% of *S*. *robusta* infection	N frogs	% of abnormality	
					Polydactyly^a^	Severe cases
Open Oxbow	*Planorbarius corneus*	1206	0.2	318	24.5	10.7
	*Planorbis planorbis*	978	0.0			
Forest Oxbow	*Planorbarius corneus*	114	0.0	211	14.7	3.8
	*Planorbis planorbis*	483	0.0			
Beaver Pond 2	*Planorbarius corneus*	690	0.2	19	36.8	10.5
	*Planorbis planorbis*	64	1.6			
Beaver Pond 1	*Planorbarius corneus*	2432	0.4	13	0.0	0.0
	*Planorbis planorbis*	570	0.0			
Anthropogenic pond	*Planorbarius corneus*	181	1.1	92	0.0	0.0
Total	*Planorbarius corneus*	4621	0.26	653	17.8	6.7
	*Planorbis planorbis*	2095	0.05			

^a^ % of abnormal tadpoles out of all tadpoles in waterbody.

## Discussion

### Diversity of trematodes from planorbid snails

*Planorbis planorbis* is a host for a minimum of 28 species of trematodes in Central Europe [14] and 20 species in Belarus [76]. In the mollusk *Pl*. *corneus* parasitized nine species in Belarus [76], nine species in Czech Republic [17], and 13 species in Central Europe [14]. In our study in one locality with seven waterbodies, we found 11 species of trematodes in *P*. *planorbis*, eight in *Pl*. *corneus* and two in *Anisus*. High taxonomical diversity of trematodes in our study (17 trematode species from five mollusk species) can be explained by high diversity of hosts in forest-steppe ecosystems as ecotone between forest and steppe ecoregions.

*Planorbarius corneus* is much more infected (mean 41.5%; 11.2–61.0%) than *P*. *planorbis* (mean 11.1%; 6.7–18.8%), which lives syntopically. However, the diversity of trematode species is higher in *P*. *planorbis* (11 species) than in *Pl*. *corneus* (8 species). There are only two-three overlaps (*H*. *asper* and *S*. *robusta*, *A*. *burti* occasionally present in *Pl*. *corneus*) in species list and each mollusk species has its own set of parasites.

It is important to note that in waterbodies of Ostrovtsovskaya Lesostep double invasions were rare in comparison with other localities studied by us (for *P*. *planorbis* 0.09% in Ostrovtsovskaya Lesostep’ *vs* 1.8% in Medvedevo Pond [77]). Possibly, parthenitae colonies of a species infecting the mollusk provide protection against co-infecting species [78, 79]. Two competing species in a snail host can feed on each other or secrete growth-inhibiting substances [80, 81]. At the same time, trematodes with rediae can feed directly on the sporocysts of other species [82] that proposed as a biological control of schistosomiasis [83, 84].

The strong dominance of *Rubenstrema exasperatum* (7.2–58.2%) can be explained by its possible high level of competitiveness. Among waterbodies, the most infected snails were found in Beaver Ponds, while in Open Oxbow the mollusk’s trematode invasion was the lowest (11.2% in *Pl*. *corneus*). This is probably due to the high infestation of the Beaver Ponds mollusks with *Rubenstrema exasperatum*, which is associated with the presence of a large number of definitive hosts–shrews from the family Soricidae [85] that often found in the Yuzhnaya River valley. Probably, the low frequency of mollusks infection with *Rubenstrema* in Open Oxbow lead to increase in *S*. *robusta* infection and, as a result, increase in abnormal rates in water frogs (10.7%; Table 8). The increase in the frequency of *Haematoloechus asper* in Open Oxbow is associated with the high density of marsh frogs, which serve as definitive hosts for the species.

Out of 24 detected species identified by helminthological dissections and cercariae screening, planorbid snails are the first intermediate hosts for 18 species (75%). Of them, 14 species parasitize the marsh frog (78% of trematodes parasitized in planorbid snails): four species as adult worms and ten on the metacercariae stage. However, not all these species of trematodes were found in planorbid mollusks. We did not find *Pharyngostomum cordatum* (Diesing, 1850) and *Haematoloechus variegatus* (Rudolphi, 1819) on cercariae stage, but detected them in frogs.

**Fig 4:**
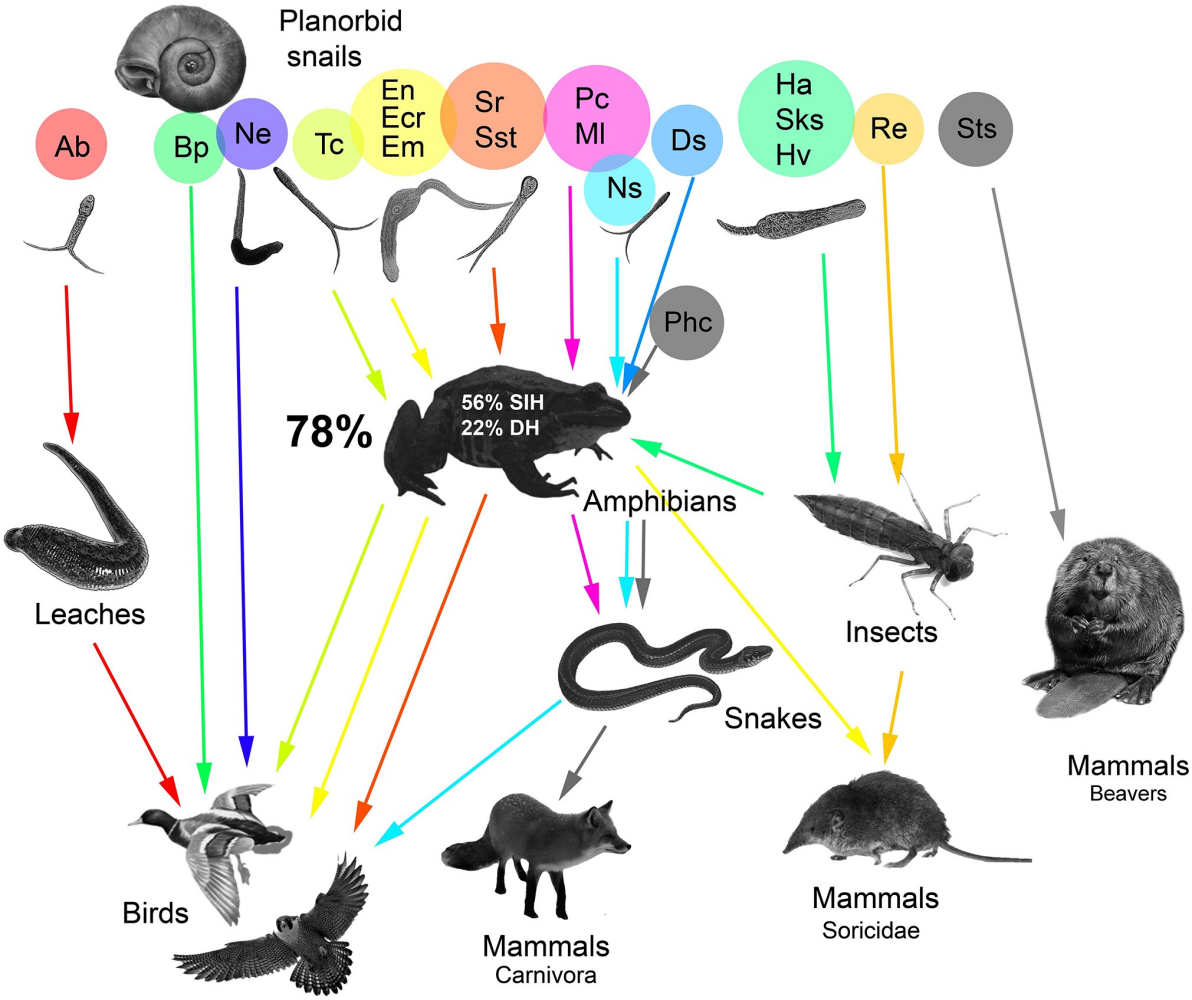
Life cycles of trematode species parasitized in planorbid snails. SIH—second intermediate host; DH—definitive host; Ab—*Australapatemon burti*, Bp—*Bilharziella polonica*, Ds—*Diplodiscus subclavatus*, Ecr—*Echinoparyphium recurvatum*, Em—*Echinostoma miyagawai*, En—*Echinostoma nasincovae*, Ha—*Haematoloechus asper*, Hv—*Haematoloechus variegatus*, Ml—*Macrodera longicollis*, Ns—*Neodiplostomum spathula*, Ne—*Notocotylus ephemera*, Pc—*Paralepoderma cloacicola*, Phc—*Pharyngostomum cordatum*, Re—*Rubenstrema exasperatum*, Sks—*Skrjabinoeces similis*, Sr—*Strigea robusta*, Sst—*Strigea strigis*, Sts—*Stichorchis subtriquetrus*, Tc—*Tylodelphys circibuteonis*.

Life cycles of detected trematodes presents in Table 9. Life cycles of trematodes parasitized in planorbid snails can be divided into nine groups (Fig 4) according to classification suggested by Kirillov et al. [85].

**Table 9:** Life cycles of trematode species found in Ostrovtsovskaya Lesostep’.

No.	Species name	FIH	SIH	DH
1	*Australapatemon burti*	Pp	Leaches	Birds
2	*Bilharziella polonica*	Pc	-	Birds
3	*Brandesia turgida*	Unknown	Unknown	Amphibians
4	*Diplodiscus subclavatus*	Pp	-	Amphibians
5	*Echinoparyphium recurvatum*	Pp	Amphibians	Birds
6	*Echinostoma miyagawai*	Pp	Amphibians	Birds
7	*Echinostoma nasincovae*	Pc	Amphibians	Mammals
8	*Gorgodera asiatica*	B	Insects	Amphibians
9	*Haematoloechus asper*	Pp, Pc	Insects	Amphibians
10	*Haematoloechus variegatus*	A	Insects	Amphibians
11	*Macrodera longicollis*	Pp	Amphibians	Reptiles
12	*Neodiplostomum spathula*	Pp	Amphibians, Reptiles	Birds
13	*Notocotylus ephemera*	Pc	-	Birds
14	*Opisthioglyphe ranae*	Ls	Amphibians	Amphibians
15	*Paralepoderma cloacicola*	Pp	Amphibians	Reptiles
16	*Pharyngostomum cordatum*	Pp	Amphibians, Reptiles	Mammals
17	*Pleurogenes claviger*	Bth	Insects	Amphibians
18	*Pleurogenoides medians*	Bth	Insects	Amphibians
19	*Prosotocus confusus*	Bth	Insects	Amphibians
20	*Rubenstrema exasperatum*	Pc	Insects	Mammals
21	*Skrjabinoeces similis*	Pp	Insects	Amphibians
22	*Stichorchis subtriquetrus*	Pp	-	Mammals
23	*Strigea robusta*	Pp, Pc	Amphibians	Birds
24	*Strigea strigis*	Pp	Amphibians, Reptiles	Birds
25	*Tylodelphys circibuteonis*	Pc	Amphibians	Birds

FIH–first intermediate host;

Pp–*Planorbis planorbis*; Pc–*Planorbarius corneus*; A–*Anisus*; B–Bivalvia; Bth–Bythiniidae; Ls–*Lymnaea stagnalis*.

SIH–second intermediate host;

DH–definitive host.

1. *Planorbid snails–mammals*. This type of life cycle is typical for *Stichorchis subtriquetrus* that parasitized in the beavers. Beavers are keystone species in forest-steppe ecosystem landscapes transformation and settled in the habitats of the Ostrovtsovskaya Lesostep’ in the 1990s. Possibly, they invade with their own parasite fauna (including *Stichorchis subtriquetrus*).

2. *Planorbid snails–birds*. This life cycle type includes species that have no second intermediate hosts: *Bilharziella polonica* and *Notocotylus ephemera*. Cercariae of *N*. *ephemera* form adolescariae on shells of mollusks or underwater substrates. The final hosts of *N*. *ephemera* are various bird species in which it causes notocotylidosis [85–88]. *Bilharziella polonica* is a species of the family Shistosomatidae that infects waterfowls [89–91]. Cercariae penetrate the bird’s skin (percutaneously) and migrate through the blood vessels to the intestinal veins, where they develop into adult worms. *Bilharziella polonica* is considered as generalists and have been reported from at least four orders of aquatic birds [89, 90]. We found this species in 0.6% of *Pl*. *corneus* and have never observed in *P*. *planorbis*. It was observed in 3.8% of the mollusk *Pl*. *corneus* in Austria [92] and 0.4% in Belarus [76].

3. *Planorbid snails–amphibians*. This form of life cycle registered for one species that have no second intermediate host and parasitized in amphibians: *Diplodiscus subclavatus*. Free-swimming cercariae of *D*. *subclavatus* are attached to the body of frog and then eaten by frogs with the skin after molting [93], but accidentally can be found in the mollusk’s gastrointestinal tract [94].

4. *Planorbid snails–amphibians–reptiles*. This variant of life cycles include species that used amphibians as the second intermediate hosts and reptiles as the definitive hosts: *Paralepoderma cloacicola* and *Macrodera longicollis*, trematodes from the family Leptophallidae [53, 85, 86]. Definitive hosts are grass snakes of the genus *Natrix* Laurenti, 1768 [85, 95, 96]. A single species of grass snakes, *Natrix natrix* (Linnaes, 1758), lives in this part of the reserve.

5. *Planorbid snails–amphibians–birds*. There are six species of trematodes that have such life strategy: *Tylodelphys circibuteonis*, *S*. *robusta*, *S*. *strigis*, *Echinoparyphium recurvatum*, *Echinostoma miyagawai*, *Neodiplostomum spathula*. We have determined by molecular analysis that furcocercariae from *Pl*. *corneus* belong to *Tylodelphys circibuteonis*. Recent research of phylogenetic relationships found uncovered cryptic speciation in the genus [63]. The life cycle of closely related species *Tylodelphys excavata* includes amphibians as the second intermediate hosts [63]. We detected *Tylodelphys circibuteonis* in mollusks by molecular analysis, but helminthological survey made it possible to detect *Tylodelphys excavata* sensu lato by morphological traits, and therefore we assumed that our individuals belong to *Tylodelphys circibuteonis*. The helminth can infect both larvae and adult amphibians. *Tylodelphys* parasitizes in the spinal canal of frogs at the stage of mesocercariae and does not form cysts retaining the ability to move [63].

Species of the genus *Strigea* are specialized in various groups of birds. There are four widespread species of *Strigea* in Europe: *S*. *strigis*, *S*. *falconis* Szidat, 1928, *S*. *sphaerula* (Rudolphi, 1803), and *S*. *robusta* [8, 97–103]. Two species of *Strigea* (*S*. *robusta* and *S*. *strigis*) were found in ponds examined. *Strigea robusta* often choose anatid birds as the final hosts, while *S*. *strigis* parasitized owls [85, 102].

*Echinostoma miyagawai* Toledo, Muñoz-Antolí et Esteban, 2000 and *Echinoparyphium recurvatum* are found in *P*. *planorbis* and at the stage of metacercariae they can infect kidney of tadpoles. The echinostomes hyperinvasion can lead to edema in tadpoles and high level of mortality [104–111]. The definitive hosts of these species are birds and mammals.

*Neodiplosthomum spathula* parasitizes intestine of birds of the genera *Aquila* Brisson, 1760 and *Falco* Linnaeus, 1758 [65]. Amphibians are the second intermediate hosts. Reptiles and mammals are the paratenic hosts. It was shown that the trematode genus *Conodiplostomum* Dubois, 1937 is paraphyletic and *C*. *spathula* (Creplin, 1829) being nested in the genus *Neodiplostomum* [65].

6. *Planorbid snails–amphibians–mammals*. Two species that use mammals as definitive hosts and amphibians as the second intermediate hosts were found: *Echinostoma nasincovae* and *Pharyngostomum cordatum* (Diesing, 1850) Ciurea, 1922. *Echinostoma nasincovae* was found in *Pl*. *corneus*, while *Echinostoma nasincovae* was probably noted earlier by various authors under the names *E*. *echinatum* or *E*. *spiniferum* as a parasite of *Pl*. *corneus*. A systematic revision of this taxon from *Pl*. *corneus* leads to describe a new species [73–75]. *Pharyngostomum cordatum* is a trematode species that infects carnivorous mammals and can lead to necrotizing enteritis in cats [112]. It uses amphibians, reptiles, birds and mammals as the reservoir hosts [85].

7. *Planorbid snails–insects–amphibians*. There are seven species of trematodes with this type of life cycle: *Gorgodera asiatica*, *Haematoloechus asper*, *Haematoloechus variegatus*, *Skrjabinoeces similis* (Looss, 1899) Sudarikov, 1950, *Pleurogenes claviger*, *Pleurogenoides medians*, *Prosotocus confusus*. *Gorgodera asiatica* parasitized Bivalvia, insects and urinary bladder of amphibians [85]. Three species of this list, *Haematoloechus asper*, *Haematoloechus variegatus*, *Skrjabinoeces similis*, have aquatic insect larvae as the second intermediate (additional) hosts. Infection of adult frogs occurs after eating of infected insect larvae. Adult worms parasitize the lungs of frogs. Four species of *Haematoloechus* (or syn. *Pneumonoeces* Loss, 1899) and *Skrjabinoeces* Sudarikov, 1950 (closely relatives): *Haematoloechus variegatus*, *Haematoloechus asper*, *Skrjabinoeces similis*, and *Skrjabinoeces breviansa* Sudarikov, 1950 were previously observed in the Middle Volga River region [85].

8. *Planorbid snails–insects–mammals*. Only one species characterized by this type of life cycle: *Rubenstrema exasperatum*. However, the species identification of the *Rubenstrema / Neoglyphe* complex was problematic: cercariae of both species *Rubenstrema exasperatum* and *Neoglyphe locellus* have very similar morphological [85] and molecular [54] traits. However, they have different morphology of adult worms. Both *Rubenstrema* and *Neoglyphe* Schaldybin, 1953 choose Soricidae Fischer, 1814 as definitive hosts, but are found in representatives of different genera (*Sorex* Linnaeus, 1758 and *Neomys* Kaup, 1829, respectively) [85].

9. *Planorbid snails–leaches–birds*. Only one species detected have leaches in its life cycle. *Australapatemon burti* was registered according to molecular analysis. The definitive hosts of the species are waterfowl birds [66, 68].

Three species recorded for the first time in the trematode fauna of Russia. *Echinostoma nasincovae* was previously registered under the names *E*. *spiniferum* (La Valette, 1855) and *E*. *echinatum* (Zeder, 1803) [14, 73–75]; *Tylodelphys circibuteonis* has not been previously reported and identified using molecular analysis; and *Australapatemon burti*, previously found in North America and the Neotropics, as well as in Central Europe and Slovakia [68], has never been found in Russia.

Some species registered in our study have epidemiological importance [85]: species of the genus *Notocotylus* Diesing, 1839 causes notocotylidosis in birds, including domestic waterfowl; *Bilharziella polonica* causes bilharziellosis in birds; *Echinostoma* Rudolphi, 1809 cause echinostomatidosis in birds and mammals; *Pharyngostomum cordatum* causes enteritis in cats and dogs [112].

### Role of planorbid snails in pathways of amphibian parasites in the anomaly P hotspot

Deformities in amphibians caused by trematodes were detected under the effects of several trematode species: *Ribeiroia ondatrae* (Price, 1931) in North America, *Acanthostomum burminis* (Bhalerao, 1926) Bhalerao, 1936 in Sri-Lanka, *Holostephanus volgensis* (Sudarikov, 1962) Vojtkova, 1966 and *Strigea robusta* in Europe and Asia [33, 37]. The most investigated variant is the *Ribeiroia ondatrae* infection in the North American amphibians [4, 13, 27, 50, 113–123]. It leads to the formation of deformities in a large number of amphibian species [13, 116, 117]. Trematode cysts are often localized around the region of the hind limbs and contribute to the development of new extra limbs on the body of developing tadpoles [113, 114]. Such deformities often reduce the locomotor activity of tadpoles and make them more accessible prey for water birds (most often the herons are the definitive hosts of the parasite *Ribeiroia ondatrae*) [116, 117]. Anomalies caused by the parasite include polymelia, sometimes polydactylia, taumelia, skin stitching at the extremities [113, 120–122]. It was shown how exposure during different stages of amphibian tadpoles leads to different malformation types [121]. At the same time, a selective effect of exposure to different species of amphibians was observed; some species were more resistant to the effects of the parasite than others [120, 122]. A synergistic interaction of the impact of several parasite species [122], as well as chemical pollution of waterbodies [123], was found, which can reduce the resistance of amphibian species in various parts of their ranges. Strong correlation between malformations and *Ribeiroia ondatrae* infection was found across many populations of amphibians in USA [13, 123].

In Eurasia, some species of trematodes cause the formation of morphological anomalies in amphibians. The trematode *Acanthostomum burminis* (Bhalerao, 1926) Bhalerao, 1936 led to amely in tadpoles [124–126] and such process increased under the action of chemical pollution of water (pesticides) [127, 128]. Additionally, *Holostephanus volgensis* (Sudarikov, 1962) Vojtkova, 1966 may influence the skeleton development: metacercariae of this species cause scoliosis in *Rana arvalis* Nilsson, 1842 tadpoles [129].

The anomaly P is one of several variants of trematode-induced malformations registered in amphibians. It was found in populations of water frogs in France, Netherlands, Morocco [34–36], and then discovered in European part of Russia [38]. The long-known anomaly has been found to be caused by a species of trematode: *Strigea robusta* lead to development of symmetrical morphological anomalies in tadpoles including polydactyly, brachymely, taumely, outgrowths, bony spikes and others [34–37]. The manipulation of the parasite is similar to *Ribeiroia*: tadpoles with the anomalies are less mobile and often become prey for near-water birds (in the case of *S*. *robusta*, ducks serve as the final host) [37]. The range of this parasite and, as a result, the place of appearance of the anomaly is much wider than it was previously known: in recent studies, they were found not only in the Penza Region of Russia, but also in the north of the Middle Volga region [69]. It has also been shown that even a small dose of cercariae can cause deformities in water frogs [70]. In many respects, such a low frequency of the trematode occurrence in planorbid mollusks, found in our study, becomes understandable: probably, an increase in the occurrence of the parasite can lead to a total infection of frogs and the extinction of the host population. The mechanisms of such containment are still unclear: they can take place at the stage of miracidia (selective infection of mollusks), or during the development of parthenits in the body of a mollusk. A high percentage of planorbid snails producing *S*. *robusta* cercariae occurs in June, and hind limbs of water frog tadpoles develop during this period.

The European water frogs of the genus *Pelophylax* Fitzinger, 1843 are the second intermediate and definitive hosts for many trematode species [8, 85, 97–100, 103]. *Pelophylax ridibundus* is parasitized by 36 species of trematodes in the Middle Volga River region [85]. Of the marsh frog trematodes recorded in the region, we found 14 species. According to the data obtained in our study, the list was supplemented by two additional species: *Strigea robusta* and *Macrodera longicollis*.

Some trematodes have amphibians in their lifecycles as the second intermediate and definitive hosts. Usually, parasites keep their final hosts alive [4, 7, 130, 131]. Although sometimes lethal hyperinvasion or toxicity of the final hosts were observed, more often parasites have mechanisms to maintain a balance in parasite-host relationships, and such stable coexistence of parasites and their hosts can take place for a long time for successful reproduction of the parasite. Host manipulation very often occurs at the metacercariae stage. The evolutionary sense is to increase the chances of delivering the parasite to its final host. Therefore, the species that form metacercariae in amphibians are of great interest. There are six genera including *Echinostoma*, *Echinoparyphium* Dietz, 1909, *Paralepoderma* Dollfus, 1950, *Macrodera* Looss, 1899, *Tylodelphys* Diesing, 1850, *Neodiplosthomum* Railliet, 1919, and *Strigea*. Some trematode species found have a negative impact on amphibian larvae and reduce their fitness. The hyperinvasion of *Echinostoma* metacercariae in kidney lead to edema and renal dysfunction in tadpoles [104–111]. The spectrum of trematode species from water bodies inhabited by planorbid mollusks with a high frequency of the anomaly P allows us to answer the question, can any trematode species lead to the formation of any morphological anomalies in amphibians? Finally, high probability to find species causing anomalies in amphibians is in the genus *Strigea* as we noted earlier [37]. One species from this list is *Strigea strigis*, which is a sister taxon for *Strigea robusta*, thus it can potentially cause malformations in amphibians and further research will shed the light on this question.

#### Institutional review board statement

The authors assert that all procedures contributing to this work comply with the ethical standards of the relevant national and institutional guides on the care and use of laboratory animals.

## Supporting information

S1 Table Occurrence of trematode cercariae in *Planorbarius corneus* mollusks during the period of 2018–2022. (XLSX)

S2 Table Occurrence of trematode cercariae in *Planorbis planorbis* mollusks during the period of 2018–2022. (XLSX)

S3 Table Occurrence of trematode cercariae in *Anisus* spp. mollusks during the period of 2018 and 2022 field seasons. (XLSX)

S4 Table A list of sequenced trematode individuals with respective hosts. (DOCX)

S1 Fig Phylogenetic relationship of trematodes based on ITS2 sequences. Maximum-likelihood phylogenetic tree of trematode species inferred using IQ-TREE with 1,000 SH-like approximate likelihood ratio test (SH-aLRT) and ultra-fast bootstrap (UFboot) replicates each. HE863950, HE863957, DQ345324, DQ345318, DQ345317 *Aspidogaster* species are used as an outgroup. Numbers at nodes indicate SH-aLRT support (≥80%)/UFboot support (≥95%); values less shown with “-”.
(JPG)
