# Supplementary material for: Diversity of trematodes from the amphibian anomaly P hotspot: Role of planorbid snails
Source: PLoS One. 2023 Mar 29;18(3):e0281740. doi: 10.1371/journal.pone.0281740 (PMC10057761; doi:10.1371/journal.pone.0281740)
Supplement: S4 Table — (DOCX) [file pone.0281740.s004.docx]

**Suppl. Table 4.** A list of sequenced trematode individuals with respective hosts.

| Species of trematodes | Host | Stage | Voucher | COI | ITS2 | 28S rRNA |
| --- | --- | --- | --- | --- | --- | --- |
| *Diplodiscus subclavatus* | *P. planorbis* | cercariae | Pp1-3 | OP715849 | OP693657 | OP709386 |
|  | *P. planorbis* | cercariae | Cer22 | OQ353086 | OQ353091 | - |
| *Notocotylus ephemera* | *Pl. corneus* | cercariae | Pc3-9 | OP721253 | OP720890 | OP720893 |
| *Stichorchis subtriquetrus* | *P. planorbis* | cercariae | Cer25 | OQ352877 | OP709262 | - |
| *Haematoloechus asper* | *Pl. corneus* | cercariae | Ro-1 | OP715855 | OP696837 | OP709769 |
|  | *P. planorbis* | cercariae | 41 | OP715854 | OP696836 | OP709768 |
|  | *Pl. corneus* | cercariae | Cer28 | OQ354224 | OQ354327 | - |
| *Haematoloechus variegatus* | *A. vortex* | cercariae | Cer23 | OQ354200 | OQ354196 | - |
| *Rubenstrema exasperatum* | *Pl. corneus* | cercariae | Plc-1 | - | MK294327 | MK585231 |
|  | *Pl. corneus* | cercariae | Cer26 | OQ354341 | OQ354215 | - |
|  | *Pl. corneus* | cercariae | Cer27 | OQ354342 | OQ354216 | - |
| *Opisthioglyphe ranae* | *P. ridibundus* | metacercariae | 10 | - | MK290404 | - |
|  | *P. ridibundus* | metacercariae | 9 | - | MK290405 | MK585344 |
|  | *P. ridibundus* | metacercariae | 8 | - | MK290406 | - |
|  | *P. ridibundus* | metacercariae | 7 | - | MK290407 | MK585343 |
|  | *P. ridibundus* | metacercariae | 6 | - | MK290408 | - |
|  | *P. ridibundus* | metacercariae | 5 | - | MK290409 | MK585342 |
|  | *P. ridibundus* | metacercariae | 4 | - | MK290410 | - |
|  | *P. ridibundus* | metacercariae | 1 | - | MK290411 | MK585341 |
|  | *P. ridibundus* | metacercariae | 101 | - | MK290412 | - |
|  | *P. ridibundus* | metacercariae | 12 | - | MK290413 | MK585346 |
|  | *P. ridibundus* | metacercariae | 11 | - | MK290414 | MK585345 |
|  | *P. ridibundus* | metacercariae | N2 | - | MK290415 | MK585340 |
| *Paralepoderma cloacicola* | *P. ridibundus* | metacercariae | 40 | OP714396 | OP704199 | OP714365 |
|  | *P. planorbis* | cercariae | Cer3 | OQ354677 | - | - |
|  | *P. planorbis* | cercariae | Pp7-4 | - | OP704200 | OP714366 |
|  | *P. planorbis* | cercariae | Pp7-2 | - | OP704201 | OP714367 |
|  | *P. ridibundus* | metacercariae | 4d | - | MK294309 | MK585218 |
|  | *P. ridibundus* | metacercariae | 10r | - | MK294310 | - |
|  | *P. ridibundus* | metacercariae | 14r | - | MK294311 | - |
| *Macrodera longicollis* | *P. planorbis* | cercariae | 61 | - | OP698001 | OP709880 |
|  | *P. ridibundus* | metacercariae | 2 | - | MK294315 | MK585199 |
| *Echinoparyphium recurvatum* | *P. planorbis* | cercariae | 54 | OP715850 | OP693486 | OP709665 |
|  | *P. planorbis* | cercariae | Pp1-2 | OP715851 | OP693487 | OP709666 |
|  | *P. planorbis* | cercariae | Pp7-3 | - | OP693488 | OP709667 |
| *Echinostoma miyagawai* | *P. planorbis* | cercariae | Pp8-1 | OP715852 | OP696595 | OP709664 |
| *Echinostoma nasincovae* | *Pl. corneus* | cercariae | Ech | OP715853 | OP694173 | OP709706 |
|  | *Pl. corneus* | cercariae | Plc-4 | - | - | MK585198 |
| *Neodiplostomum spathula* | *P. planorbis* | cercariae | 56 | OP715856 | OP720892 | OP720891 |
| *Australapatemon burti* | *P. planorbis* | cercariae | 50 | OP715848 | OP693490 | OP709375 |
| *Strigea strigis* | *P. planorbis* | cercariae | 58 | OP715857 | OP704220 | OP714364 |
| *Strigea robusta* | *Pl. corneus* | cercariae | Isolate 1 | - | MT075803 | MT075841 |
|  |  |  | Isolate 2 | - | MT075804 | MT075842 |
| *Tylodelphys circibuteonis* | *Pl. corneus* | cercariae | Pc6-7 | OP715858 | OP704224 | OP714394 |
|  | *Pl. corneus* | cercariae | TE-1 | - | - | OP714395 |
| *Bilharziella polonica* | *Pl. corneus* | cercariae | Pc3-4 | - | OP693618 | OP709387 |
